# Associations between adverse childhood experiences and insomnia: The moderating role of social capital in a three-year longitudinal study from the Japan Gerontological Evaluation Study

**DOI:** 10.1016/j.pmedr.2025.103319

**Published:** 2025-11-21

**Authors:** Nao Tomono, Masamichi Hanazato, Katsunori Kondo, Kazushige Ide, Atsushi Nakagomi

**Affiliations:** a Graduate School of Medical and Pharmaceutical Sciences, Chiba University, 1-8-1, Inohana, Chuo-ku, Chiba-shi, Chiba 260-8670, Japan; b Center for Preventive Medical Sciences, Chiba University, 1-33, Yayoicho, Inage-ku, Chiba-shi, Chiba 263-8522, Japan; c Institute for Health Economics and Policy, Association for Health Economics Research and Social Insurance and Welfare, 1-21-19, Toranomon, Minato-ku, Tokyo 105-0001, Japan

**Keywords:** Insomnia, Sleep, Adverse childhood experiences, Adversity, Individual social capital, Longitudinal study, Older adults

## Abstract

**Objective:**

Adverse childhood experiences (ACEs) are linked to sleep disturbances later in life. Social capital may promote better sleep among older adults, but its moderating role remains unclear. We examined whether social capital modifies the ACE–insomnia association.

**Methods:**

Longitudinal data from the Japan Gerontological Evaluation Study (2013–2016, Japan) included 8890 adults aged 65 years and older. Insomnia in 2016 was assessed with the Athens Insomnia Scale. ACEs were measured retrospectively in 2016, and social capital in 2013 across civic participation, social cohesion and reciprocity. Poisson regression estimated relative risks (RR) of insomnia. Effect-measure modification (EMM) was evaluated on multiplicative and additive scales.

**Results:**

More ACEs were associated with a higher probability of insomnia (per +1 ACE: RR = 1.19; 95 % CI = 1.15, 1.23). Higher civic participation, social cohesion, and reciprocity were inversely associated with insomnia (RRs = 0.87–0.94). EMM was limited: civic participation showed a small interaction in the amplifying direction (multiplicative RR = 1.04; *p* = 0.03), whereas social cohesion and reciprocity showed no interaction.

**Conclusions:**

Social capital showed limited modification of the ACE–insomnia association. Social cohesion remained independently protective, while civic participation slightly amplified the association. Fostering cohesive, supportive communities may promote sleep health in later life, including among those with ACE histories.

## Highlights

• Higher adverse childhood experience count is associated with higher insomnia risk.
• Civic participation, social cohesion, and reciprocity were inversely associated.
• Effect modification by social capital was limited.
• Civic participation amplified insomnia risk with adverse childhood experiences.
• Fostering social cohesion may benefit sleep health in older adults.

## Graphical abstract

**Figure:**
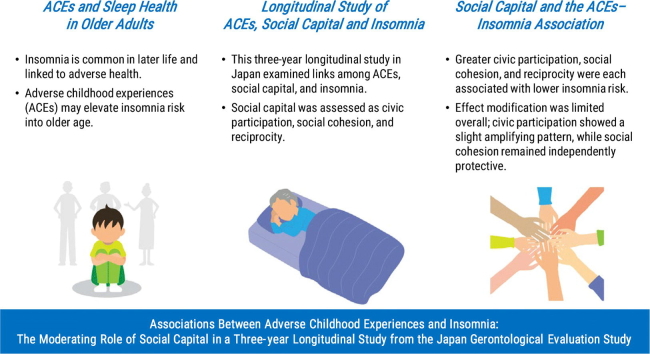

## 1 Introduction

Sleep is a key lifestyle factor for health. It is linked to hypertension, depression, dementia, and higher mortality (Cappuccio et al., 2010). Age-related changes increase sleep decline in older adults. In Japan, approximately one in three older adults has a sleep disorder (Watanabe et al., 2020). Improving sleep in older adults is a public health priority that may reduce healthcare costs (Hafner et al., 2017).

There is growing interest in adverse childhood experiences (ACEs) as risk factors for sleep disorders (Kajeepeta et al., 2015). ACEs—such as abuse and household dysfunction—are a major public health concern. At least one ACE is reported by 63.9 % of U.S. adults (Swedo et al., 2023) and 67 % of older adults in Japan (Matsukura et al., 2023). ACEs are linked to depression, ischemic heart disease, premature mortality and sleep disorders (Kajeepeta et al., 2015). Epidemiological studies suggest that individuals exposed to ACEs are more likely to experience insomnia symptoms in adulthood. Emotional neglect and emotional abuse have been consistently associated with insomnia, and the number and diversity of ACEs appear to increase the risk and severity of sleep disturbances (Bader et al., 2007; Wang et al., 2016). Biological evidence supports this view: childhood adversity has been associated with fragmented rapid eye movement sleep and increased nighttime arousals (Bader et al., 2007; Insana et al., 2012), as well as with epigenetic changes in stress-related genes, which heighten lifetime stress reactivity (Van Someren, 2021). ACEs may contribute to insomnia through biological and social pathways—disrupting neurobiological regulation of arousal while fostering lifelong exposure to stress, maladaptive coping, and re-victimization (Kajeepeta et al., 2015; Van Someren, 2021).

In this context, social capital may be an important factor. As proposed by the sociologist Bourdieu, social capital was originally conceptualized as resources embedded in social relations. It was later popularized in public health by Putnam as features of social organization—such as trust, norms, and networks—that can facilitate collective action. It is commonly organized along cognitive dimensions (e.g., trust, reciprocity) and structural dimensions (e.g., social participation, networks) (Kawachi and Berkman, 2000). Higher social capital has been associated with better mental and physical health outcomes, including lower risks of depression, cardiovascular disease, and sleep problems (Monk et al., 2003; Yang et al., 2022). These findings suggest that social capital may serve as a general protective resource for maintaining health in later life.

Beyond this main effect, social capital may buffer the link between ACEs and adverse health outcomes. Strong social networks can lower risks of mental illness (Afifi et al., 2016) and dementia (Tani et al., 2021) among those with ACE histories. However, social capital may not function as a protective factor. ACEs may impair cognitive and emotional development by altering brain structures, including reduced hippocampal and prefrontal volumes—regions involved in emotion processing (Teicher et al., 2016). Neurodevelopmental changes may hinder interpersonal functioning and limit the use of social capital, reducing its buffering potential. Individuals with ACE histories may face heightened risks of intergenerational violence and prolonged exposure to violent environments (Koga et al., 2024). Since social relationships can reproduce such adversity, attention must be paid to the potentially negative dimensions—or the “dark side”—of social capital in these contexts (Portes, 1998). Social capital may either buffer or, in some contexts, amplify the impact of ACEs on health. To our knowledge, no study has examined whether social capital modifies the ACE–insomnia association among older adults. We aimed to (1) assess the association between ACEs and insomnia and (2) test effect measure modification (EMM) by social capital. Following a previous study (Saito et al., 2017), we assessed social capital using three dimensions—civic participation, social cohesion, and reciprocity. We hypothesized that the moderating roles of these dimensions on the ACE–insomnia association could differ (Kawachi and Berkman, 2000).

## 2 Methods

### 2.1 Study design

**Fig. 1:**
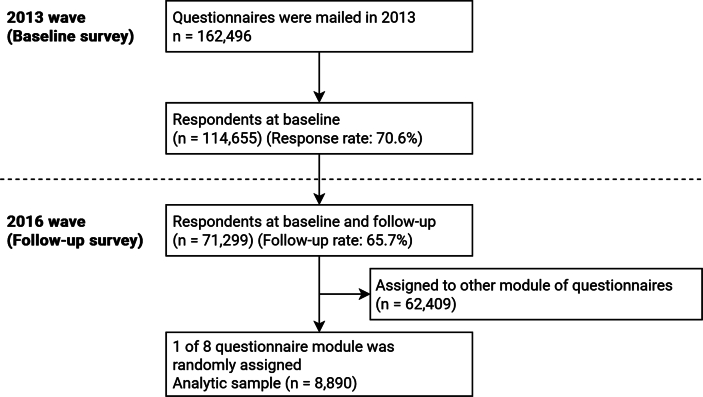
Participant flow and exclusion criteria in the longitudinal study among older adults, Japan, 2013–2016.

This three-year longitudinal study used data from the Japan Gerontological Evaluation Study (JAGES) (Kondo and Rosenberg, 2018). The baseline survey (Oct 2013–Jan 2014) mailed questionnaires to 162,496 adults (≥65 years) in 25 municipalities, yielding 114,655 responses (70.6 %); the follow-up (Nov 2016–Jan 2017) included 71,299 of 108,488 respondents (65.7 %). The questionnaire included core items and one of eight modules (Kondo et al., 2018); one module on insomnia and ACEs was randomly distributed. The final analysis included 8890 participants (Fig. 1; Table S1). Returning the questionnaire implied informed consent. This study was approved by the ethics committee of Chiba University (No. 2493). Procedures complied with the Declaration of Helsinki.

### 2.2 Measures

#### 2.2.1 Outcome: Insomnia

Insomnia symptoms in the last month were assessed as the dependent variable in the 2016 survey. The Japanese version of the Athens Insomnia Scale was used to assess insomnia (Okajima et al., 2013). The total score for the eight items ranged from 0 to 24, with scores ≥6 indicating insomnia.

#### 2.2.2 Exposures: ACEs and moderator: Social capital

We conceptualized social capital as a moderator of the ACE–insomnia relationship. In 2016, participants retrospectively reported whether they had experienced ACEs by responding to the following question: “Did you experience any of the following before the age of 18? (Yes/No); 1) parental loss (either or both parents due to war or other causes), 2) parental divorce, 3) mother or father suffering a mental illness, 4) your father was violent toward your mother, 5) hit so hard by your mother/father that it caused an injury, 6) felt loved by your parents (reverse coded), 7) harsh words/insults directed toward you by your mother/father, and 8) childhood poverty”. Although not in the original ACEs study (Felitti et al., 1998), a recent study includes childhood poverty (Matsukura et al., 2023). We summed ACEs and categorized them as 0, 1, 2, or ≥ 3. ACE questions were also asked in 2013, but combining those data with social capital and covariates would have cut the analytic sample to about one-eighth because the ACE and insomnia modules differed in 2016. As ACEs are retrospective and unlikely to change, we used the 2016 responses.

This study assessed individual-level social capital in 2013 using three validated scales: civic participation, social cohesion, and reciprocity (Saito et al., 2017). Civic participation was quantified as the total number of groups (sports, hobby, cultural, volunteer, or skill-teaching groups) in which the individual took part more than once per month. Social cohesion was measured by the number of positive responses (“moderately or strongly agree” on a five-point Likert scale) to three statements concerning trust in neighbors, perceived helpfulness of community members, and attachment to the community. Reciprocity was defined by the count of affirmative answers to questions about the availability of someone to listen to one's concerns, the act of listening to others' concerns, and having support during a short-term illness. Each scale was standardized to z-scores.

#### 2.2.3 Covariates and potential confounders

All covariates were measured in 2013 and adjusted as potential confounders. Demographic/SES: age (65–69, 70–74, 75–79, ≥80), gender, equivalent income (<2.0, 2.0–3.99, ≥4.0 million yen; <US$20,000, US$20,000–39,900, and ≥ US$40,000 per year, based on the 2013 average exchange rate), education (<9, 10–12, ≥13 years), marital status (married, widowed, divorced, never married), and employment (working, retired, never worked). Neighborhood context: population density (≤1000, 1001–1500, 1501–4000, ≥4001 persons/km^2^; Statistics Bureau definition of Densely Inhabited District). Height (five-cm categories) was included as a proxy for childhood nutrition and socioeconomic status. Health/behavioral factors: BMI (<18.5, 18.5–24.9, ≥25.0), diseases under treatment (hypertension, heart, respiratory, gastrointestinal, urinary, chronic pain, cancer), smoking (never, past, current), drinking (never, past, current), and Instrumental Activities of Daily Living (dependent 0–4 vs independent 5) (Tanimoto et al., 2012; Taylor et al., 2007; Yang et al., 2022). Depressive symptoms were assessed with the 15-item Geriatric Depression Scale (0–4 none, 5–9 mild, 10–15 severe) (Sugishita et al., 2017).

We used social capital measured in 2013 and insomnia and ACEs measured in 2016 because module allocation differed by wave and cross-wave combinations substantially reduced sample size (Supplementary Tables S2–S3).

### 2.3 Statistical analysis

Descriptive analysis examined characteristics. We used modified Poisson regression to calculate relative risks (RR) and 95 % confidence intervals (CI) for insomnia in 2016 in relation to ACEs and each social capital dimension. The analysis was conducted in the following order: Model 1 adjusted for age, gender, income, education, marital status, employment status, population density, height, body mass index, disease, smoking status, drinking status and IADL; Model 2 adjusted for depressive symptoms to assess confounding by mental health; Model 1 omits them to avoid overadjustment given depression's potential mediation between ACEs and insomnia. We tested EMM—not mediation—on multiplicative (RR) and additive (absolute risk difference, AME) scales (Mize, 2019). Multiplicative EMM used modified Poisson models with robust variance and ACE × social-capital terms. Additive EMM used marginal standardization to obtain predicted risks and AMEs per +1 ACE at −1 SD and + 1 SD, summarized as ΔAME (+1 SD minus −1 SD). We stratified by low (−1 SD) and high (+1 SD) social capital. To assess potential selection bias, we conducted sensitivity analyses using stabilized inverse probability weights with propensity scores from logistic regression on the covariates.

We used multiple imputation (multivariate normal method, 20 datasets). Analyses used Stata/MP 18 (StataCorp LLC).

## 3 Results

### 3.1 Descriptive statistics

Table 1 presents the characteristics by ACE histories: no ACEs (*n* = 2842; 32 %), one ACE (*n* = 2948; 33 %), two ACEs (*n* = 1564; 18 %), and three or more ACEs (*n* = 557; 6 %). Insomnia was reported by 17 % of those with no ACEs and 31 % of those with three or more. Social capital scores were lower with more ACEs: civic participation −0.03 to −0.26; social cohesion 0.11 to −0.27; reciprocity 0.12 to −0.31 (means from no ACEs to three or more ACEs). Regarding socioeconomic status, income <2 million yen was seen in 35 % with no ACEs and 53 % with three or more; <10 years of education was reported in 28 % and 61 %, respectively.

**Table 1:** Demographic characteristics among older adults by number of adverse childhood experiences, Japan, 2013–2016 (*n* = 8890).

Characteristics	Adverse Childhood Experience									
	Total		0		1		2		≥3	
	(n = 8890)		(n = 2842)		(n = 2948)		(n = 1564)		(n = 557)	
	N	%	N	%	N	%	N	%	N	%
Insomnia										
No	5866	66	2071	73	2005	68	996	64	317	57
Yes	1814	20	487	17	597	20	388	25	174	31
Missing	1210	14	284	10	346	12	180	12	66	12
Social capital										
Civic Participation, Mean, SD	−0.10	0.96	−0.03	0.98	−0.09	0.96	−0.14	0.94	−0.26	0.89
Social Cohesion, Mean, SD	0.01	1.00	0.11	0.94	−0.01	1.01	−0.05	1.03	−0.27	1.06
Reciprocity, Mean, SD	0.01	0.99	0.12	0.73	0.02	0.99	−0.02	1.02	−0.31	1.46
Gender										
Male	4044	45	1084	38	1384	47	806	52	335	60
Female	4846	55	1758	62	1564	53	758	48	222	40
Age (in years)										
65–69	2757	31	1026	36	896	30	442	28	193	35
70–74	2922	33	839	30	1012	34	571	37	201	36
75–79	1938	22	550	19	647	22	365	23	110	20
≥80	1273	14	427	15	393	13	186	12	53	10
Annual income (in millions of yen)										
Low (<2.00)	3598	40	985	35	1202	41	738	47	293	53
Middle (2.00–3.99)	2862	32	1050	37	960	33	489	31	135	24
High (≥4.00)	847	10	371	13	270	9	110	7	42	8
Missing	1583	18	436	15	516	18	227	15	87	16
Education										
Less than 10 years	3646	41	786	28	1170	40	826	53	337	61
10–12 years	3345	38	1245	44	1155	39	510	33	140	25
More than 13 years	1757	20	779	27	582	20	199	13	69	12
Missing	142	2	32	1	41	1	29	2	11	2
Marital status										
Married	6541	74	2133	75	2222	75	1162	74	394	71
Widowed	1697	19	538	19	521	18	287	18	98	18
Divorced	241	3	67	2	80	3	38	2	26	5
Never married	173	2	60	2	50	2	33	2	14	3
Missing	238	3	44	2	75	3	44	3	25	4
Employment status										
Working	2068	23	676	24	704	24	344	22	143	26
Retired	5153	58	1608	57	1747	59	998	64	320	57
Never worked	924	10	363	13	274	9	117	7	52	9
Missing	745	8	195	7	223	8	105	7	42	8
Population Density										
<1000 population/km^2^	2066	23	595	21	694	24	365	23	144	26
1000–1500 population/km^2^	1807	20	583	21	600	20	314	20	103	18
1500–4000 population/km^2^	2511	28	801	28	814	28	460	29	150	27
≥4000 population/km^2^	2506	28	863	30	840	28	425	27	160	29
Height										
Short (male: <155, female: <145 cm)	753	8	206	7	224	8	136	9	55	10
Middle-short (male: 155–159.9; female: 145–149.9 cm)	1864	21	556	20	630	21	330	21	112	20
Middle (male: 160–164.9; female: 150–154.9 cm)	3058	34	984	35	1017	34	543	35	185	33
Middle-tall (male: 165–169.9; female: 155–159.9 cm)	2053	23	703	25	684	23	372	24	132	24
Tall (male: ≥170; female: ≥160 cm)	1162	13	393	14	393	13	183	12	73	13
Body mass index										
Underweight (<18.5)	554	6	189	7	191	6	86	5	30	5
Normal (18.5–24.9)	6075	68	2014	71	2018	68	1040	66	363	65
Overweight (≥25.0)	1929	22	557	20	643	22	375	24	145	26
Missing	332	4	82	3	96	3	63	4	19	3
Disease										
No	2937	33	997	35	968	33	528	34	170	31
Yes	5424	61	1677	59	1803	61	957	61	352	63
Missing	529	6	168	6	177	6	79	5	35	6
Smoking status										
None	6538	74	2216	78	2131	72	1106	71	345	62
Past	1381	16	368	13	496	17	271	17	125	22
Current	850	10	237	8	280	9	170	11	79	14
Missing	121	1	21	1	41	1	17	1	8	1
Drinking status										
None	5258	59	1807	64	1715	58	874	56	269	48
Past	404	5	91	3	147	5	76	5	30	5
Current	3117	35	926	33	1045	35	597	38	251	45
Missing	111	1	18	1	41	1	17	1	7	1
IADL										
Independent (≥5)	7346	83	2463	87	2439	83	1263	81	417	75
Dependent (0–4)	1313	15	319	11	431	15	269	17	124	22
Missing	231	3	60	2	78	3	32	2	16	3
Depression										
None (<5)	5735	65	2061	73	1937	66	939	60	279	50
Mild (5–9)	1474	17	352	12	491	17	331	21	152	27
Severe (10–15)	267	3	43	2	85	3	60	4	43	8
Missing	1414	16	386	14	435	15	234	15	83	15

Note: Civic participation was measured as the number of types of community group activities in which respondents participated at least monthly. Social cohesion was assessed from three items on perceived trust, mutual help, and attachment to the neighborhood. Reciprocity was assessed from items on both receiving and providing emotional or instrumental support. Social capital variables (civic participation, social cohesion, reciprocity) were standardized (mean = 0, SD = 1). *n* = 979 had missing data for ACEs. Percentages may not sum to 100 % because of rounding or missing values.

Abbreviations: ACEs, Adverse Childhood Experiences; IADL, Instrumental Activities of Daily Living.

### 3.2 Association between ACEs, insomnia, and social capital

Table 2 shows the relative risk (RR) of insomnia by cumulative ACEs from multivariable Poisson regression with robust error variance. In the primary model without depressive-symptom adjustment (Model 1), ACEs were associated with insomnia (RR = 1.19; 95 % CI: 1.15, 1.23). Higher civic participation (per +1 SD: RR = 0.92; 95 % CI: 0.87, 0.98), social cohesion (RR = 0.87; 95 % CI: 0.83, 0.92), and reciprocity (RR = 0.94; 95 % CI: 0.89, 0.98) were each associated with a lower probability of insomnia. On the multiplicative scale, only civic participation showed evidence of interaction with ACEs (ACE × civic participation: RR = 1.04; *p* = 0.03), whereas interactions with social cohesion (RR = 1.02; *p* = 0.26) and reciprocity (RR = 1.01; *p* = 0.58) were not significant. On the additive scale, the ΔAME of ACEs comparing +1 SD with −1 SD of civic participation was +0.02 (i.e., the AME of ACEs on insomnia was 2 percentage points larger at +1 SD vs −1 SD; *p* = 0.08). At the reference level of each social capital dimension (standardized mean = 0), the predicted probability of insomnia was approximately 0.25 (25 %), indicating that any additive-scale effect modification by civic participation was small in absolute terms.

**Table 2:** Poisson regression analysis of adverse childhood experiences and insomnia among older adults, and the moderating role of social capital, Japan, 2013–2016 (n = 8890).

Exposure /Moderator	Main effect on insomnia			Multiplicative EMM			Additive EMM		
	RR	95 % CI	Sig.	RR	*P*-value	Sig.	ΔAME	P-value	Sig.
Model 1									
Number of ACEs	1.19	1.15, 1.23	**	NA	NA	NA	NA	NA	NA
Civic participation	0.92	0.87, 0.98	**	1.04	0.03	*	+0.02	0.08	
Social cohesion	0.87	0.83, 0.92	**	1.02	0.26		0.00	0.94	
Reciprocity	0.94	0.89, 0.98	**	1.01	0.58		0.00	0.71	
Model 2									
Number of ACEs	1.14	1.11, 1.19	**	NA	NA	NA	NA	NA	NA
Civic participation	0.97	0.91, 1.03		1.04	0.05	*	+0.02	0.04	*
Social cohesion	0.93	0.88, 0.98	*	1.02	0.15		+0.01	0.28	
Reciprocity	0.98	0.93, 1.02		1.01	0.44		0.00	0.53	

Note: ACEs were modeled as a count (per +1 adverse experience). Civic participation was measured as the number of types of community group activities in which respondents participated at least monthly. Social cohesion was assessed from three items on perceived trust, mutual help, and attachment to the neighborhood. Reciprocity was assessed from items on both receiving and providing emotional or instrumental support. Social capital variables (civic participation, social cohesion, reciprocity) were standardized (mean = 0, SD = 1); coefficients for these variables are interpreted per 1-SD increase. AME denotes the absolute change in the predicted probability of insomnia per one-unit (or 1-SD) increase in the exposure, holding other variables constant. ΔAME denotes the difference in AME between +1 SD and − 1 SD (i.e., the additive-scale index of EMM). For reference, at the standardized mean (0) of each social capital dimension, the model-predicted probability of insomnia was approximately 25 % (i.e., the absolute risk at the reference level). Model 1: adjusted for age, gender, income, education, marital status, employment status, population density, height, body mass index, chronic disease, smoking, drinking, and instrumental activities of daily living. Model 2: Model 1 + depression.

Abbreviations: ACEs, Adverse Childhood Experiences; RR, Relative Risk; CI, Confidence Interval; Sig., Significance * *p* < 0.05, ** *p* < 0.01; EMM, Effect Measure Modification; AME, Average Marginal Effect; NA, Not Applicable.

**Fig. 2:**
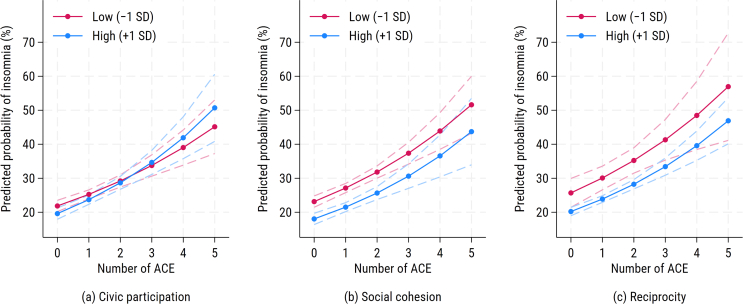
Predicted probability of insomnia by number of adverse childhood experiences and levels of social capital among older adults, Japan, 2013–2016 (Model 1: unadjusted for depressive symptoms). Panels show (a) civic participation, (b) social cohesion, and (c) reciprocity. Civic participation was measured as the number of types of community group activities in which respondents participated at least monthly. Social cohesion was assessed from three items on perceived trust, mutual help, and attachment to the neighborhood. Reciprocity was assessed from items on both receiving and providing emotional or instrumental support. Lines depict model-based predicted probabilities from modified Poisson regression with robust variance, marginalized over covariates. For each panel, “Low” and “High” indicate the moderator set to −1 SD and + 1 SD of its standardized (z-score) value, respectively; dashed lines indicate 95 % confidence intervals. The y-axis is displayed in percentage units. Abbreviations: ACE, adverse childhood experience.

**Fig. 3:**
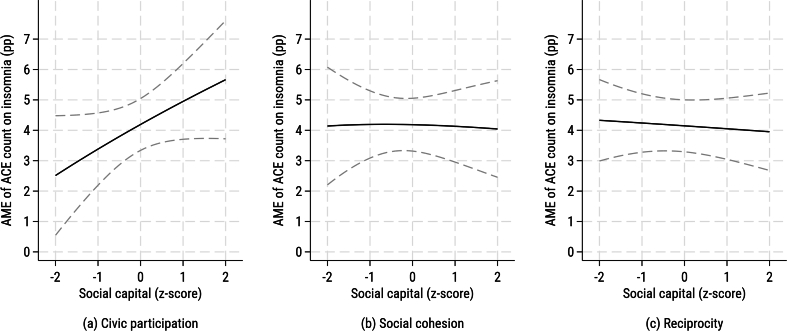
Average marginal effects of adverse childhood experiences on insomnia by dimension of social capital among older adults, Japan, 2013–2016 (Model 1: unadjusted for depressive symptoms). Panels show (a) civic participation, (b) social cohesion, and (c) reciprocity. Civic participation was measured as the number of types of community group activities in which respondents participated at least monthly. Social cohesion was assessed from three items on perceived trust, mutual help, and attachment to the neighborhood. Reciprocity was assessed from items on both receiving and providing emotional or instrumental support. Lines show point estimates and shaded areas 95 % confidence intervals. Abbreviations: ACE, adverse childhood experience; AME, average marginal effect.

Fig. 2, Fig. 3 show that the ACE–insomnia slope is slightly steeper at higher civic participation (+1 SD) than at lower civic participation (−1 SD), whereas the lines for social cohesion and reciprocity are nearly parallel; the corresponding AMEs of ACEs on insomnia are almost flat across the range of each social capital dimension, with only a small upward trend at higher civic participation, indicating that effect modification was small and limited to civic participation. With additional adjustment for depressive symptoms (Model 2), the ACE–insomnia association persisted (RR = 1.14; 95 % CI: 1.11, 1.19). Main effects for civic participation and reciprocity attenuated to the null, while social cohesion remained inversely associated (RR = 0.93; 95 % CI: 0.88, 0.98). The ACE × civic participation interaction was borderline on the multiplicative scale (RR = 1.04, *p* = 0.05) and significant on the additive scale (ΔAME = +0.02, *p* = 0.04), whereas interactions for the other two dimensions remained non-significant. Subgroup analyses at −1 SD vs +1 SD yielded comparable RRs (Supplementary Table S4), indicating limited moderation. Robustness checks using inverse probability weights (Supplementary Table S5) showed similar patterns.

## 4 Discussion

To our knowledge, this is the first longitudinal study to examine whether social capital modifies the association between ACEs and insomnia among older adults. In line with prior research, a higher number of ACEs was associated with a higher probability of insomnia, whereas higher social capital showed protective main associations. Evidence for EMM was limited: civic participation suggested a small amplifying effect, whereas social cohesion and reciprocity showed no clear moderation.

### 4.1 Association between ACEs and insomnia

Our findings indicate that the cumulative number of ACEs was positively associated with the probability of insomnia. This result is consistent with previous research showing that childhood trauma and adversity have long-term impacts on adult health outcomes (Felitti, 1998) and that multiple ACEs are associated with higher risks of sleep problems (Yu et al., 2022). Mechanisms may involve persistent hyperarousal, as proposed in the model of trauma-induced chronic insomnia and circadian dysregulation (Werner et al., 2021). Additionally, individuals with ACEs may be exposed to stressors that degrade sleep (Greenfield et al., 2011). These findings support the involvement of both biological and social mechanisms, as discussed in the Introduction.

### 4.2 Examining social capital as a potential moderator

Among the three dimensions of social capital, only civic participation showed a small amplification of the ACE–insomnia association; social cohesion and reciprocity showed no moderation on either scale. Plausibly, in those with ACE histories, heightened stress reactivity and trait-like hyperarousal (Werner et al., 2021) intersect with participation-related social strain to worsen sleep (Chung, 2017), slightly steepening the slope at higher participation. For social cohesion and reciprocity, the ACE–insomnia slope was largely invariant. Positive pathways of social cohesion (trust, perceived safety) (Kawachi and Berkman, 2000) may be offset by conformity pressures (Villalonga-Olives and Kawachi, 2017) and reputational concerns (Huang et al., 2025), yielding a net null on moderation. More broadly, the “dark side” of social capital (Portes, 1998) can add stress depending on context, while ACE-related alterations in stress-regulatory circuits may limit the buffering capacity of social resources (Teicher et al., 2016). Notably, social cohesion retained a consistent protective main association. Practically, when promoting participation, emphasize psychological safety and quality, and in parallel strengthen community cohesion, especially for those with ACE histories.

Depression may act as both a confounder and a mediator. Because ACEs can induce depressive symptoms that affect sleep and social capital, overadjustment is possible. Therefore, Model 1 excluded depression to estimate the total ACE effect, whereas Model 2 included it to test robustness to residual confounding.

Our findings complement prior dementia results in the same cohort (Tani et al., 2021): that study reported EMM, whereas we observe a protective main association for social cohesion with limited modification. Differences likely reflect outcome time scales and pathophysiology (dementia: long-term cumulative; insomnia: shorter-term psychological/behavioral), as well as measurement timing and effect scales. Future work should test pathways from insomnia to cognitive decline.

### 4.3 Implications and future directions

This study suggests directions for future research. Prospective and experimental studies should test whether enhancing individual and community-level social cohesion prevents insomnia. One avenue is the development of “third places,” informal public settings outside of home and work, such as cafes, parks, and libraries. Parks—one type of third place—have been reported to strengthen social cohesion (Kaźmierczak, 2013). In Japan, evaluations of “*Kayoi-no-ba*” (community gathering places) are advancing, with evidence that they promote healthy longevity—particularly among socioeconomically disadvantaged groups (Ide et al., 2025). Strengthening social connections may enhance social cohesion and lower insomnia risk among older adults with ACE histories.

### 4.4 Strengths and weaknesses

Strengths of this study include the simultaneous analysis of ACEs and three social capital dimensions within a longitudinal design that preserves temporal ordering between social capital and insomnia. Several limitations should be noted. First, ACEs were assessed retrospectively and may be subject to recall bias. Prior studies suggest recall is reliable (Hardt and Rutter, 2004); underreporting may occur, but overestimation is unlikely. Second, we used ACEs from 2016; although concordance with 2013 responses was high (82–99 %), bias from different assessment times cannot be ruled out. Third, measures were self-reported, introducing common method bias. Fourth, the sample comprised healthy older adults, suggesting survival bias and possible underestimation. Fifth, being observational, unmeasured confounding cannot be ruled out. Sixth, baseline insomnia in 2013 was unavailable, precluding incident analyses; thus, we estimate associations, not causal effects. Seventh, although the sample was large, interactions require more power; post hoc power was 60–99 % across models. Some null or borderline findings may reflect limited power, and estimates should be interpreted given small effect sizes and their dependence on scale (additive vs. multiplicative) and specifications. Eighth, the use of older data is a limitation. We analysed the 2013–2016 waves because they are the only ones that include both ACEs and insomnia with a sample size large enough for adequately powered analyses (Supplementary Tables S2–S3). Analyses limited to the more recent 2016–2019 waves would have insufficient sample size and low statistical power, and later waves do not measure ACEs. As adverse childhood experiences and social capital are relatively stable over periods under ten years, this lag is unlikely to materially change the observed associations, but it may restrict generalizability to the current older population.

## 5 Conclusions

In summary, this study using data from regions in Japan found that ACEs were associated with higher insomnia risk in older adults, while higher levels of individual social capital appeared to be associated with lower insomnia risk. Evidence for EMM was limited: social cohesion and reciprocity showed no clear moderation, whereas civic participation exhibited a small difference in the amplifying direction. Future research should assess whether community-level strategies to strengthen social cohesion and psychologically safe forms of individual participation may promote sleep health among older adults, including those with ACE histories.

## CRediT authorship contribution statement

**Nao Tomono:** Writing – review & editing, Writing – original draft, Visualization, Methodology, Formal analysis, Conceptualization. **Masamichi Hanazato:** Writing – review & editing, Validation, Resources, Methodology, Conceptualization. **Katsunori Kondo:** Writing – review & editing, Supervision, Resources, Funding acquisition. **Kazushige Ide:** Writing – review & editing, Validation, Resources, Methodology, Conceptualization. **Atsushi Nakagomi:** Writing – review & editing, Validation, Supervision, Resources, Methodology, Conceptualization.

## Declaration of generative AI and AI-assisted technologies in the writing process

During the preparation of this work, the authors used ChatGPT-5 in to improve language and readability. After using this tool, the authors reviewed and edited the content as needed and take full responsibility for the content of the publication.

## Funding

This study was supported by Grant-in-Aid for Scientific Research (19K02200, 20H00557, 20H03954, 20K02176, 20K10540, 20K13721, 20K19534, 21H03153, 21H03196, 21K02001, 21K10323, 21K11108, 21K17302, 21K17308, 21K17322, 22H00934, 22H03299, 22J00662, 22J01409, 22K01434, 22K04450, 22K10564, 22K11101, 22K13558, 22K17265, 22K17409, 23K16320, 23H00449, 23H03117, 23K19793, 23K16349, 23H00060, 25K01387) from the JSPS (10.13039/501100001691Japan Society for the Promotion of Science), Health Labour Sciences Research Grants (19FA1012, 19FA2001, 21FA1012,22FA2001, 22FA1010, 22FG2001, 24LA1002), Research Funding for Longevity Sciences from 10.13039/501100007312National Center for Geriatrics and Gerontology (21−20), 10.13039/501100009028Research Institute of Science and Technology for Society (JPMJOP1831) from the Japan Science and Technology for Society (JST), a grant from Japan Health Promotion & Fitness Foundation, contribution by the Department of Active Ageing, 10.13039/100012833Niigata University Graduate School of Medical and Dental Sciences (donated by Tokamachi city, Niigata), TMDU priority research areas grant, and National Research Institute for Earth Science and Disaster Resilience. The views and opinions expressed in this article are those of the authors and do not necessarily reflect the official policies or positions of the respective funding organizations.

## Declaration of competing interest

The authors declare that they have no known competing financial interests or personal relationships that could have appeared to influence the work reported in this paper.

## Data Availability

Researchers may request anonymized data from the JAGES office (https://www.jages.net/data_application/) with proposal review, approval, and a signed data use agreement per JAGES guidelines.

## Appendix A Supplementary data

Supplementary material. Image 1
