# Supplementary material for: Associations between adverse childhood experiences and insomnia: The moderating role of social capital in a three-year longitudinal study from the Japan Gerontological Evaluation Study
Source: Prev Med Rep. 2025 Nov 21;60:103319. doi: 10.1016/j.pmedr.2025.103319 (PMC12702049; doi:10.1016/j.pmedr.2025.103319)
Supplement: Supplementary file 1 — Supplementary material. [file mmc1.docx]

Associations Between Adverse Childhood Experiences and Insomnia:

The Moderating Role of Social Capital in a Three-year Longitudinal Study from the Japan Gerontological Evaluation Study

Supplemental material

**Table S1. Population and number of participants in the surveyed municipality, Japan, 2013–2016**

| Municipality Name | Prefecture | Population | Number of participants | Percentage | Cumulative percentage |
| --- | --- | --- | --- | --- | --- |
| A Town | Yamanashi | Less than 10 thousand | 20 | 0.2% | 0.2% |
| B Town | Hokkaido | Less than 10 thousand | 92 | 1.0% | 1.3% |
| C Town | Mie | Less than 10 thousand | 116 | 1.3% | 2.6% |
| D Town | Hokkaido | Less than 10 thousand | 90 | 1.0% | 3.6% |
| E Town | Hokkaido | 10–50 thousand | 144 | 1.6% | 5.2% |
| F Town | Kumamoto | 10–50 thousand | 108 | 1.2% | 6.4% |
| G Town | Aichi | 10–50 thousand | 219 | 2.5% | 8.9% |
| A City | Nagasaki | 10–50 thousand | 256 | 2.9% | 11.8% |
| H Town | Aichi | 10–50 thousand | 303 | 3.4% | 15.2% |
| B City | Yamanashi | 10–50 thousand | 298 | 3.4% | 18.5% |
| I Town | Aichi | 10–50 thousand | 506 | 5.7% | 24.2% |
| C City | Miyagi | 10–50 thousand | 450 | 5.1% | 29.3% |
| J Town | Aichi | 10–50 thousand | 119 | 1.3% | 30.6% |
| D City | Aichi | 50–100 thousand | 638 | 7.2% | 37.8% |
| E City | Aomori | 50–100 thousand | 268 | 3.0% | 40.8% |
| F City | Aichi | 50–100 thousand | 273 | 3.1% | 43.9% |
| G City | Aichi | 50–100 thousand | 237 | 2.7% | 46.5% |
| H City | Aichi | 50–100 thousand | 202 | 2.3% | 48.8% |
| I City | Aichi | 100 thousand–1 million | 235 | 2.6% | 51.5% |
| J City | Aichi | 100 thousand–1 million | 594 | 6.7% | 58.1% |
| K City | Aichi | 100 thousand–1 million | 871 | 9.8% | 67.9% |
| L City | Chiba | 100 thousand–1 million | 345 | 3.9% | 71.8% |
| M City | Niigata | 100 thousand–1 million | 383 | 4.3% | 76.1% |
| N City | Aichi | More than 300 thousand | 1,475 | 16.6% | 92.7% |
| O City | Kanagawa | More than 1 million | 648 | 7.3% | 100.0% |

**Table S2. Availability of sleep, adverse childhood experiences, and social capital measures across Japan Gerontological Evaluation Study waves, 2010, 2013, 2016, and 2019**

| Survey Year | Sleep measures | ACEs measures | Social capital measures |
| --- | --- | --- | --- |
| 2010 | Include, Sleep quality (single-item measure); module | Not included | Include; full sample |
| 2013 | Not included | Include; module | Include; full sample |
| 2016 | Include, Athens Insomnia Scale; module | Include; module | Include; full sample |
| 2019 | Include, Athens Insomnia Scale; module | Not included | Include; full sample |

Notes. “Include” indicates the measure was available in that wave; “module” indicates distribution to a random subsample. AIS denotes the Athens Insomnia Scale (2016, 2019). Abbreviations: ACEs, Adverse Childhood Experiences; JAGES, Japan Gerontological Evaluation Study.

**Table S3. Alternative wave combinations, measurement timing, and sample sizes**

| Dataset | Sample size | Measurement wave | | | | Remarks |
| --- | --- | --- | --- | --- | --- | --- |
|  |  | Insomnia | ACEs | Social capital | Covariates |  |
| 2013-2016 | 8,890 | 2016 | 2016 | 2013 | 2013 | Analytic Dataset |
| 2010-2013-2016 | 1,038 | 2016 | 2016 | 2013 | 2010 | Reduction in sample size  (insufficient power for interaction analyses) |
| 2016-2019 | 1,115 | 2019 | 2016 | 2016 | 2016 | Reduction in sample size  (insufficient power for interaction analyses) |

Note: “Measurement wave” columns indicate the wave used for each construct in the dataset. “Reduction in sample size” flags combinations with limited power due to module misalignment. Abbreviations: ACEs, Adverse Childhood Experiences; JAGES, Japan Gerontological Evaluation Study.

**Table S4. Associations between adverse childhood experiences and insomnia in subgroup analyses by social capital dimensions among older adults, Japan, 2013–2016 (n=8,890)**

| Moderator | Subgroup | N | RR | 95% CI | Sig. |
| --- | --- | --- | --- | --- | --- |
| Civic Participation | Low (−1 SD) | 4,476 | 1.16 | 1.11, 1.21 | ** |
|  | High (+1 SD) | 4,353 | 1.19 | 1.13, 1.26 | ** |
| Social cohesion | Low (−1 SD) | 4,607 | 1.16 | 1.12, 1.21 | ** |
|  | High (+1 SD) | 4,258 | 1.20 | 1.13, 1.27 | ** |
| Reciprocity | Low (−1 SD) | 839 | 1.16 | 1.07, 1.26 | ** |
|  | High (+1 SD) | 8,030 | 1.18 | 1.13, 1.22 | ** |

Note. RR: Relative Risk; CI: Confidence Interval; Sig.: significance, * p < 0.05, ** p < 0.01; Models were adjusted for covariates, including the other social capital variables not used for stratification, age, gender, income, education, marital status, employment status, population density, height, body mass index, chronic disease, smoking, drinking, and instrumental activities of daily living. Civic participation was measured as the number of types of community group activities in which respondents participated at least monthly. Social cohesion was assessed from three items on perceived trust, mutual help, and attachment to the neighborhood. Reciprocity was assessed from items on both receiving and providing emotional or instrumental support.

**Table S5. Inverse probability weighting–based robustness checks of associations between adverse childhood experiences and insomnia and effect modification by social capital, Japan, 2013–2016 (n=8,890)**

| Exposure / moderator | Main effect on insomnia | | | Multiplicative EMM | | | Additive EMM | | |
| --- | --- | --- | --- | --- | --- | --- | --- | --- | --- |
|  | RR | 95% CI | Sig. | RR | P-value | Sig. | ΔAME | P-value | Sig. |
| Model 1 |  |  |  |  |  |  |  |  |  |
| Number of ACEs | 1.19 | 1.15, 1.23 | ** | NA | NA | NA | NA | NA | NA |
| Civic participation | 0.92 | 0.87, 0.98 | ** | 1.04 | 0.03 | * | +0.02 | 0.07 |  |
| Social cohesion | 0.87 | 0.83, 0.92 | ** | 1.02 | 0.29 |  | 0.00 | 0.90 |  |
| Reciprocity | 0.93 | 0.89, 0.98 | ** | 1.01 | 0.56 |  | 0.00 | 0.72 |  |
| Model 2 |  |  |  |  |  |  |  |  |  |
| Number of ACEs | 1.15 | 1.10, 1.19 | ** | NA | NA | NA | NA | NA | NA |
| Civic participation | 0.97 | 0.91, 1.03 |  | 1.04 | 0.05 | * | +0.02 | 0.04 | * |
| Social cohesion | 0.93 | 0.88, 0.99 | * | 1.02 | 0.19 |  | +0.01 | 0.33 |  |
| Reciprocity | 0.97 | 0.93, 1.02 |  | 1.01 | 0.42 |  | 0.00 | 0.52 |  |

Note: ACEs were modeled as a count (per +1 adverse experience). Civic participation was measured as the number of types of community group activities in which respondents participated at least monthly. Social cohesion was assessed from three items on perceived trust, mutual help, and attachment to the neighborhood. Reciprocity was assessed from items on both receiving and providing emotional or instrumental support. Social capital variables (civic participation, social cohesion, reciprocity) were standardized (mean = 0, SD = 1); coefficients for these variables are interpreted per 1-SD increase. AME denotes the absolute change in the predicted probability of insomnia per one-unit (or 1-SD) increase in the exposure, holding other variables constant. ΔAME denotes the difference in AME between +1 SD and −1 SD (i.e., the additive-scale index of EMM). For reference, at the standardized mean (0) of each social capital dimension, the model-predicted probability of insomnia was approximately 25% (i.e., the absolute risk at the reference level). Model 1: adjusted for age, gender, income, education, marital status, employment status, population density, height, body mass index, chronic disease, smoking, drinking, and instrumental activities of daily living. Model 2: Model 1 + depression.

Abbreviations: ACEs, Adverse Childhood Experiences; RR, Relative Risk; CI, Confidence Interval; Sig., Significance * p < 0.05, ** p < 0.01; EMM, Effect Measure Modification; AME, Average Marginal Effect; NA, Not Applicable.
